# Rapid and sensitive electrochemiluminescence detection using easily fabricated sensor with an integrated two-electrode system†

**DOI:** 10.1039/d3ra07298c

**Published:** 2024-01-19

**Authors:** Haojun Yuan, Baihui Liang, Ping Yang, Zhiwei Yang, Xinyi Cao, Yangbo Wu, Jie Zou, Qinghui Jin, Wanlei Gao

**Affiliations:** a College of Information Science and Engineering, Ningbo University Ningbo 315211 Zhejiang China gaowanlei@nbu.edu.cn jinqinghui@nbu.edu.cn; b Healthy & Intelligent Kitchen Engineering Research Center of Zhejiang Province Ningbo 315336 Zhejiang China; c Ningbo Fotile Kitchen Ware Company Ningbo 315336 Zhejiang China

## Abstract

The electrochemiluminescence (ECL) behavior of a tri(2,2′-bipyridyl)ruthenium(ii) (Ru(bpy)_3_^2+^)/tripropylamine (TPrA) system was investigated in sensor chips with two kinds of integrated two-electrode systems, which included screen-printed electrodes (SPE) and physical vapor deposition (PVD) electrodes. Firstly, under excitation with an optimal transient potential (TP) within 100 ms, the ECL assay could be carried out on the microchips using an Au & Au electrode system, emitting strong and stable light signal. Secondly, on the PVD chip, the ECL intensity initiated by optimal TP was eight times stronger than the peak light signal emitted by the linear sweep voltammetry model. Finally, the logarithmic ECL intensities exhibited a linear increase with the logarithmic concentrations of Ru(bpy)_3_^2+^ in both the SPE and PVD chips without any reference electrode (RE). Typically, the integration of an interdigital two-electrode system in the microchip significantly enhanced the ECL sensitivity of Ru(bpy)_3_^2+^ because the large relative area between the working electrode (WE) and counter electrode (CE) achieved a highly efficient mass transfer. This improvement enabled the establishment of a reliable linear relationship across a wide concentration range, spanning from 1 pM to 1 μM (*R*^2^ = 0.998). Therefore, the exceptional ECL response of the Ru(bpy)_3_^2+^/TPrA system on microfluidic chips using a two-electrode system and the TP excitation model has been demonstrated. This suggests that ECL chips without a RE have broad potential for the rapid and sensitive detection of multiple targets.

## Abstract 2

This work presents the exceptional ECL response of the tri(2,2′-bipyridyl)ruthenium(ii) (Ru(bpy)_3_^2+^)/tripropylamine (TPrA) system on microfluidic chips embedded with integrated electrode systems using the transient potential excitation model.

## Graphical abstract

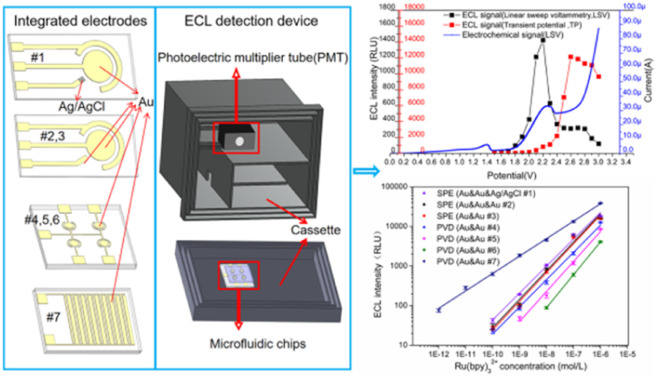

## 1. Introduction

Electrochemiluminescence (ECL) was discovered almost a century ago. ECL involves an electrochemical redox reaction occurring at the electrode surface, causing typical molecules to become excited and reach a higher energy state. Light signals are generated when the excited molecules return to their original state.^1^ ECL provides a higher signal to noise ratio compared to fluorescent-based detection. As a result, ECL-based analytical techniques offer the following advantages: (1) superior sensitivity and linear detection range, (2) excellent temporal and spatial resolution due to electrochemical potential dependency, and (3) simple and fast measurement.^2–4^ With these advantages, ECL has been extensively used in various fields including biosensing, chemical detection, imaging, and so on over the past 30 to 40 years.^5–7^

Recent developments in materials have further expanded the application of ECL technology.^8–10^ The original ECL luminophore, tris(2,2′-bipyridine) ruthenium(ii) dichloride, also known as Ru(bpy)_3_^2+^, only produced fluorescence in aprotic electrolytes.^11^ However, the introduction of co-reactants, such as amines, alcohols, and amino acids, has enabled ECL to occur in aqueous solutions, leading to a breakthrough in the scale of detection.^12^ Additionally, the luminol and H_2_O_2_ ECL system, a classic ECL system, exhibits excellent ECL activity.^13^ Although Ru(bpy)_3_^2+^ and luminol, along with their co-reactants, have limited applications due to difficulties in labeling analytes, the use of nano-materials such as CdSe quantum dots (QDs) has shown promise in revealing ECL activity, and can be easily modified with biomolecules.^12,14^

In addition to the development of new ECL systems, extensive research has been conducted on the electrode material, surface status and structure to improve ECL signal strength.^15–19^ For example, Neužil's group presented a nanostructured gold amalgam microelectrode array with a high surface-to-volume ratio, which enhanced the electrical current for ECL.^11^ In ECL experiments, large-sized, Ag/AgCl electrode filled with saturated KCl solution and platinum (Pt) electrodes were used as reference and counter electrodes, respectively. With the continuous development of microfluidic technology and microelectro-mechanical systems (MEMS) technology, many micro-electrode systems have been integrated into microfluidic chips for the sensitive detection of biomolecules.^20–22^ Satienperakul's group developed a simple 3D-printed platform with a three-electrode system, which was used to detect sibutramine in dietary supplements, demonstrating the broad potential for micro-electrode systems in ECL-based biosensor applications.^23^ Although current microchips utilize a three-electrode system to achieve ECL reaction, three-electrode system increases the complexity of multi-detection applications on a single microchip.

Bipolar electrode (BPE) based chip, requires only two driving electrodes to exert potential over many BPE arrays, presenting an excellent option for realizing multiplex ECL assays.^24^ Compared to traditional three-electrode system, BPE-ECL chips significantly simplify detecting unit and operation. For the two poles of BPE in the same electrolyte solution, the target will react on the driving electrode, bringing a strong background signal and reducing the sensitivity of detection. Xu's group designed an Indium Tim Oxide (ITO) single-electrode electrochemical system (SEES) for high-throughput ECL tests, which was free from the ECL background problem in BPE system.^25^ ECL assays in each cell on one SEES chip were initiated by the same pair wires, therefore light signals for each detection cell only can be measured simultaneously by charge coupled device (CCD), not photomultiplier tube (PMT).

If a two-electrode system without a RE is able to replace the three-electrode system and ensure a stable electrochemical potential for light emission, it may be readily to allow for a two-electrode system array (at least four units) in one ECL microchip, making them suitable for the simultaneous detection of multiple parameters. Currently, there is a lack of detailed research on the impact of integrated electrode structures and constituents on the efficiency of ECL assay. Gold is a preferred metal for sensor fabrication due to its simplicity, stability, and potential for surface chemistry *via* thiol cross-linkers. To investigate the performance of different microchip configurations, simple sensor chips with integrated electrodes were fabricated from gold using screen printing and physical vapor deposition (PVD) to create a two-electrode system, respectively. And the ECL performance of Ru(bpy)_3_^2+^/tripropylamine (TPrA) system was studied on different chips. Under the excitation with an optimal transient potential (TP), ECL assay could be carried out on the chips with two-electrode systems. The effect of electrodes' size and buffer's ionic concentration on the ECL behavior of Ru(bpy)_3_^2+^/TPrA was confirmed with PVD microchip. Furthermore, there was a strong linear correlation between the logarithmic ECL intensity and the concentration of Ru(bpy)_3_^2+^ in all chips, without including any RE. These findings suggest that a microchip utilizing a two-electrode system for ECL is a promising possibility.

## 2. Experimental

### 2.1 Reagents and solutions

All reagents were of analytical grade and used directly without further purification. Tris(2,2′-bipyridyl)dichloro ruthenium(ii) hexahydrate were purchased from Sigma-Aldrich (Shanghai) Trading Co., Ltd. PBS solution with concentration of 0.1 M (10× PBS, pH = 7.0) were purchased from Sangon Biotech (Shanghai) Co., Ltd. Absolute ethanol was purchased from Shanghai Titan Scientific Co., Ltd. Tripropylamine (TPrA) was purchased from Shanghai Yien Chemical Technology Co., Ltd. Ultra-pure deionized water (18.25 MΩ, LD-UPW-1 Water Purification System, Shanghai Leading Water Treatment Equipment Co., Ltd) was used for all solutions.

A stock solution of Ru(bpy)_3_^2+^ (0.1 mM) was prepared by dissolving the appropriate quality of tris(2,2′-bipyridyl)dichloro ruthenium(ii) hexahydrate in the 0.01 M PBS buffer (pH 7.0). A series of Ru(bpy)_3_^2+^ reagent solution ranging from 1 pM to 1 μM were prepared with the appropriate dilution of the Ru(bpy)_3_^2+^ stock solution in the PBS buffer. TPrA reagent solution for ECL assay was prepared with adding the appropriate amount of TPrA solution in the working solution of Ru(bpy)_3_^2+^.

### 2.2 Design and fabrication of ECL microchips

**Fig. 1:**
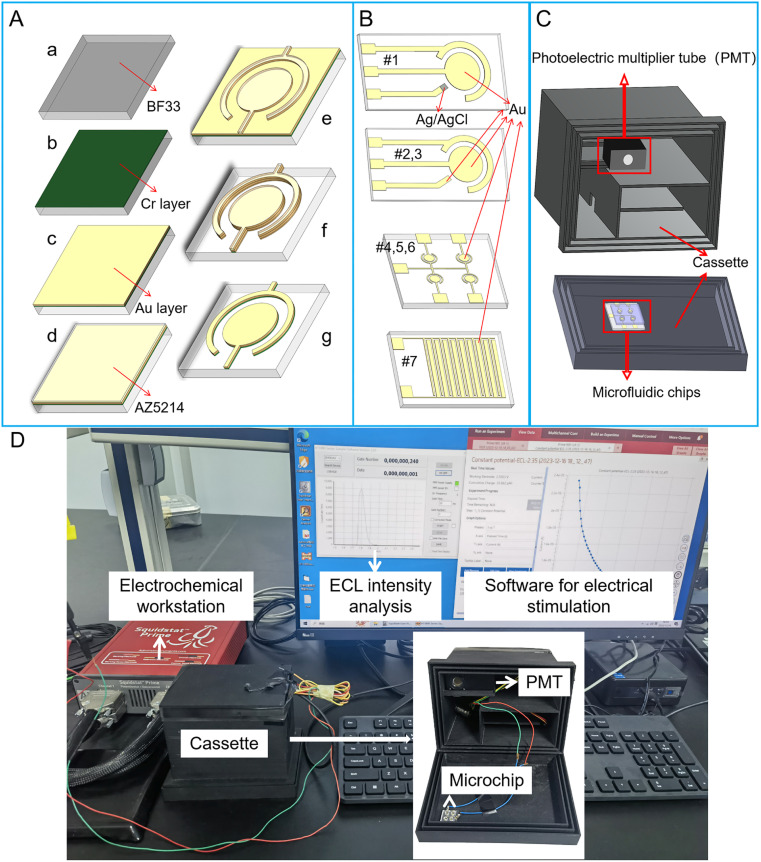
The fabrication and design of ECL microchips and instruments for ECL detection. (A) Fabrication process of PVD ECL microchips. (B) ECL microchips with different electrodes. (#1) SPE microchip with two Au electrodes and Ag/AgCl electrode. (#2) SPE microchip with three Au electrodes. (#3) SPE microchip with two Au electrodes. (#4–6) PVD chips with four circular WEs and one shared CE. (#7) PVD chip with interdigital electrode system. (C) Schematic diagram of cassette required for ECL assays on microchips. A PMT for capturing single photon was placed in a 3D printed cassette. There was a slot at the cover of the cassette for locating the microchip, whose detection area were aligned to the photosensitive area of the PMT. (D) The image of ECL detection instruments, which included cassette embedded with a PMT, electrochemical workstation and PC for result readout.

Two kinds of screen-printed electrode (SPE) microchips were purchased from Beijing Mingtai Jiaxin Technology Co., Ltd, which include a three-electrode system. The working electrode (WE) and counter electrode (CE) for the two SPE microchips are gold (Au) electrodes. The reference electrode (RE) for SPE microchip #1 and #2 are Ag/AgCl and Au electrode, respectively (Fig. 1B#1 and #2). SPE microchip #3 with two-electrode system includes an Au WE and Au CE, whose sizes are the same with that of SPE microchip #2 (Fig. 1B#2). Structure sizes of SPE microchips are listed in Table 1. The radius of the WE in the three SPE microchips is 1.5 mm. The distance between WE and CE is 750 μm. The whole size of SPE microchip is 12 mm × 45 mm.

**Table 1:** Relevant parameters of seven microchips, including three kinds of screen-printed electrode (SPE) microchips and four kinds of physical vapor deposition (PVD) microchips

Structure no.	Type	Electrode system	Working electrode size^a^	The area of working electrode	Distance^a^	Microchip size
#1	SPE	Three-electrode (Au & Au & Ag/AgCl) (Fig. 1B-#1)	*R* = 1.5 mm	7.065 mm^2^	*D* = 750 μm	12 mm × 45 mm
#2	SPE	Three-electrode (Au & Au & Au) (Fig. 1B-#2)	*R* = 1.5 mm	7.065 mm^2^	*D* = 750 μm	12 mm × 45 mm
#3	SPE	Two-electrode (Fig. 1B-#3)	*R* = 1.5 mm	7.065 mm^2^	*D* = 750 μm	12 mm × 45 mm
#4	PVD	Two-electrode (Fig. 1B-#4)	*R* = 900 μm	2.543 mm^2^	*D* = 200 μm	13 mm × 13 mm
#5	PVD	Two-electrode (Fig. 1B-#5)	*R* = 800 μm	2.010 mm^2^	*D* = 200 μm	13 mm × 13 mm
#6	PVD	Two-electrode (Fig. 1B-#6)	*R* = 650 μm	1.327 mm^2^	*D* = 200 μm	13 mm × 13 mm
#7	PVD	Interdigital two-electrode (ten fingers) (Fig. 1B-#7)	*W* = 200 μm	6 mm^2^	*D* = 150 μm	6 mm × 8 mm
			*L* = 3 mm			

a *R* means the radius of circular electrode. *W* means the width of finger in interdigital electrodes. *L* means the length of finger in interdigital electrodes. *D* means the distance between working electrode and counter electrode.

Four kinds of microchips with two-electrode system were fabricated through physical vapor deposition (PVD) method. Structure sizes of PVD microchips are listed in Table 1. Among PVD microchips, three of them contain electrode array, which includes four circular WE and one shared CE (Fig. 1B#4, 5 and 6). For PVD microchips #4, #5 and #6, the radius of WE are 900 μm, 800 μm and 650 μm, respectively. The distance between WE and CE in PVD microchips #4, #5 and #6 are all 200 μm. The whole size of PVD microchips #4, #5 and #6 are 13 mm × 13 mm. In addition, a PVD microchip #7 with interdigital electrode system was developed, which contains ten fingers (Fig. 1B#7). The width and length of every finger is 200 μm and 3 mm. The spacing between each finger in interdigital electrode system is 150 μm. The whole size of PVD microchip #7 is 6 mm × 8 mm. Additional five PVD microchips of various sizes, labeled as #8, #9, #10, #11 and #12, were listed in Table S1.†

The fabrication process of PVD microchips is shown in Fig. 1A. Firstly, the BF33 glass substrate was cleaned using a plasma degumming machine (Fig. 1Aa). A 10 nm chromium layer (Cr) as an adhesion layer and a 200 nm Au layer as electrode layer were added to the wafer, respectively, *via* sputter coating (Fig. 1Ab and c). The wafer was then coated with AZ5214 and micro-structures were patterned through the lithography process (Fig. 1Ad and e). Next, the metal (Au and Cr) layers were removed from the wafer *via* ion beam etching (IBE) (Fig. 1Af). The electrode structures were finally immersed in acetone, which was agitated using ultrasound, to dissolve the photoresist for a duration of 5 minutes (Fig. 1Ag).

For each microchip, a PDMS well was designed based on the area of the electrode system. The diameters of the well for SPE microchip, PVD microchip and PVD microchip with interdigital electrode system were 5 mm, 1.5 mm and 5 mm, respectively. The fabrication process of PDMS well was as follows: a PDMS mixture (in a 10 : 1 ratio of base and curing agent) was poured on the top of the silicon mold, degassed in vacuum and cured in an 80 °C oven for 1 h. After curing, the PDMS was cut into pieces as the size of microchip and punched with needle to form a well. The tailored PDMS chip and microchip were aligned together through plasma treatment. At last, the device was incubated on a hot plate at 120 °C for 8 h to strengthen the bonding between the PDMS chip and microchip.

### 2.3 ECL equipment

The ECL measurement system for micro-chip was developed in-house. The electrochemical reaction was initiated by the linear sweep voltammetry (LSV) or transient potential (TP) model using an electrochemical workstation (Gamry Reference 600+, USA). The light from ECL assay was detected using a photomultiplier tube (PMT, H11890, Hamamatsu electron, Japan), which was placed in a 3D printed cassette (Guangzhou Xingyou Technology Co., Ltd). The PMT module was capable of detecting single photons and counting the transistor–transistor logic (TTL) pulses with a selected sampling frequency. There was a slot at the cover of the cassette for locating the microchip, whose detection area was aligned to the photosensitive area of the PMT (Fig. 1C). As shown in Fig. 1D, the wires for connecting microchips to the electrochemical workstation are arranged in the cassette to avoid light. An output ECL signal was observed continually and exported to a personal computer (PC). A software on PC was used for showing the intensity of ECL light.

### 2.4 Device operation and ECL assay

Prior to each ECL assay, microchip was placed at the slot in the cassette. And the electrodes for each chip were connected with the electrochemical workstation through wires. The ECL equipment was connected to PC through USB connector. Sulfuric acid solution (0.5 M, 10 μL) was added to the well of the microchip for activating electrodes. The activation process was triggered by cyclic voltammetry three times in a range of −0.6–1.5 V, with a scan rate of 100 mV s^−1^. Afterwards, the chip was sequentially rinsed with water and ethanol. When the chip was dry, a mixture of 20 μL Ru(bpy)_3_^2+^ with a certain concentration and 30 mM TPrA were added into the well on the microchip. For detection of different concentration of TPrA, the sample was a mixture of 20 μL TPrA with a certain concentration and 10 μM Ru(bpy)_3_^2+^. The samples for ECL assay were all prepared at room temperature (∼25 °C). The measurement of ECL assay was commonly conducted at room temperature of approximately 25 °C as reported in study.^1^ For the ECL measurement, the cassette was closed and still for 3 min. For LSV model, the ECL reaction was initiated by LSV stimulation. The ECL light was captured once every 10 ms using PMT module during the whole process of LSV scanning. For TP model, the ECL reaction was initiated when a voltage was applied by the electrochemical workstation for 100 ms. At the same time, the ECL light was captured once every 10 ms using PMT module. The ECL intensity for each test was calculated according to the formula, Δ*V* = *V* − *V*_0_, where *V*_0_ represents the ECL peak intensity of the sample before TP stimulation, and *V* is the ECL peak intensity during the voltage stimulation.

## 3. Results and discussion

### 3.1 ECL behavior of Ru(bpy)_3_^2+^/TPrA system on microchip

**Fig. 2:**
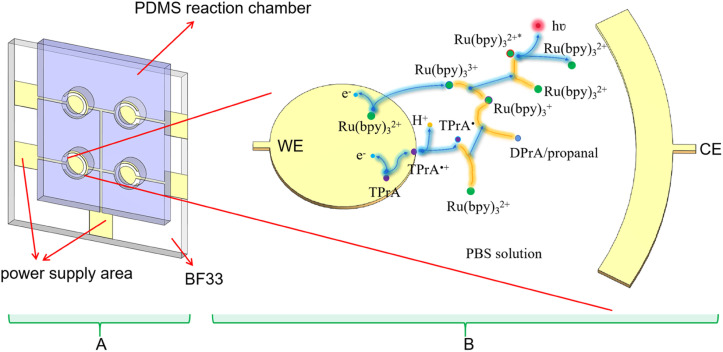
Schematic diagram of reaction principle of Ru(bpy)_3_^2+^ on ECL microchip. (A) Schematic representation of an ECL microchip, which contains four electrode units. (B) Reaction scheme of Ru(bpy)_3_^2+^/TPrA system on electrode in the microchip.

Currently Ru(bpy)_3_^2+^ is an important ECL luminophore, the electrochemical and ECL properties of which have been studied extensively. With TPrA as a co-reactant, the ECL reaction of Ru(bpy)_3_^2+^ can occurred in aqueous solutions. The mixture of Ru(bpy)_3_^2+^ and TPrA was added in the PDMS well of the microchip (Fig. 2A). Each electrode unit on the PVD microchip has an Au WE and a shared Au CE. The oxidative-reduction process of Ru(bpy)_3_^2+^ and TPrA is carried out on the surface of WE in our proposed microchip (Fig. 2B). The reaction equation shown in Fig. 2B is as follows:

1 TPrA → TPrA˙^+^ + e^−^

2 TPrA˙^+^ → TPrA + H^+^

3 Ru(bpy)_3_^2+^ → Ru(bpy)_3_^3+^ + e^−^

4 Ru(bpy)_3_^2+^ + TPrA˙ → Ru(bpy)_3_^+^ + DPrA/propanal

5 Ru(bpy)_3_^3+^ + Ru(bpy)_3_^+^ → Ru(bpy)_3_^2+^* + Ru(bpy)_3_^2+^

6 Ru(bpy)_3_^2+^* → Ru(bpy)_3_^2+^ + *hν*

where ‘*h*’ is Planck's constant, ‘*ν*’ is the photon frequency. ‘*hν*’ represents photon energy.

First the oxidation of TPrA is occurred on the surface of the WE (eqn (1)). At the same time, Ru(bpy)_3_^2+^ loses an electron to form Ru(bpy)_3_^3+^ (eqn (3)). Then Ru(bpy)_3_^2+^ is reduced by TPrA radical to turn into Ru(bpy)_3_^+^. Ru(bpy)_3_^+^ continued to reduce Ru(bpy)_3_^3+^ to Ru(bpy)_3_^2+^ and Ru(bpy)_3_^2+^* (eqn (5)). When the excited-state molecule returns to its ground state, light of 620 nm is produced (eqn (6)). The ground-state Ru(bpy)_3_^2+^ can go on to react with TPrA.^26^ Therefore, if TPrA is excess in the solution, this reaction process can continue to produce light.

### 3.2 Effect of reference electrode and voltage stimulation model on ECL behavior of Ru(bpy)_3_^2+^/TPrA system on microchip

**Fig. 3:**
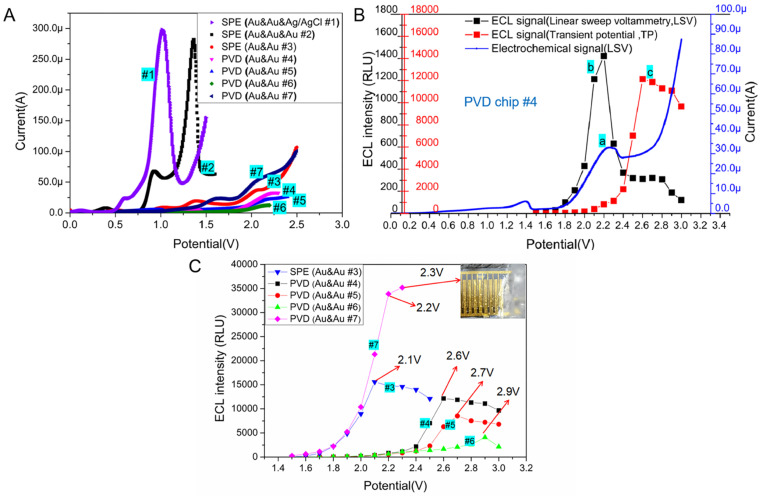
(A) Voltammograms obtained at different microchips (SPE chip #1, #2 and #3, PVD chip #4, #5, #6 and #7) in PBS solution (0.01 M, pH 7.0) containing 0.1 μM Ru(bpy)_3_^2+^ and 30 mM TPrA. Scan rate was 100 mV s^−1^ (B) ECL response (the black line labeled b) and simultaneous voltammograms (the blue line labeled a) obtained at the PVD chip #4 with Au & Au-electrode system in PBS solution (0.01 M, pH 7.0) containing 0.1 μM Ru(bpy)_3_^2+^ and 30 mM TPrA. Scan rate was 100 mV s^−1^. The left *y*-axis whose label was black represented the intensity of ECL signal under LSV scanning. The left *y*-axis whose label was red represented the intensity of ECL signal under transient potential (TP) excitation. The red line represented ECL signal at the PVD chip #4 when the chip was applied by TP in the range of 1.5 V to 3.0 V. (C)ECL intensity from 0.1 μM Ru(bpy)_3_^2+^ and 30 mM TPrA in PBS solution (0.01 M, pH 7.0), achieved in microchip with two-electrode system. The dots in each line represented ECL intensity during the measurement under different TP in the range of 1.5 V to 3.0 V. The inset showed the electrolysis of water was occurred on the electrodes of PVD #7 at 2.3 V.

Generally, the oxidative-reduction process of Ru(bpy)_3_^2+^ and TPrA is achieved by three-electrode electrochemical devices. To evaluate the electrochemical behavior of Ru(bpy)_3_^2+^/TPrA system on microchips, the electrocatalytic oxidation current was measured on two different three-electrode systems of SPE chips with the mix of 0.1 μM Ru(bpy)_3_^2+^ and 30 mM TPrA. For the three-electrode system with an Ag/AgCl RE, the oxidation peak potential of the electrochemistry (EC) process was at ∼1.0 V (see curve #1 in Fig. 3A). On the SPE chip with an Au RE, the oxidation peak of Ru(bpy)_3_^2+^ appeared at ∼1.3 V (see curve #2 in Fig. 3A). The peak currents of EC process for the two SPE chips with three electrodes differed only by 16 μA. Without a RE in SPE chip, the peak potential shifted to 2.1 V and the peak current sharply decreased to 38 μA (see curve #3 in Fig. 3A). With the size of WE decreased in the PVD chips, the peak potential shifted slightly to the right, reaching about 2.2 V (see curve #4, #5 and #6 in Fig. 3A). Compared with circular WE, the peak potential for the oxidation of Ru(bpy)_3_^2+^ on the interdigital electrodes decreased to 2.1 V and the peak current increased to 56.1 μA. The peak current for chip #7 was the largest among the two-electrode based devices (see curve #7 in Fig. 3A).

The ECL behavior of the Ru(bpy)_3_^2+^ and TPrA system was studied on the circular Au WE with a radius of 900 μm, which was located in PVD chip #4. During linear sweep voltammetry (LSV) process, the potential reached 2.2 V, the current of Ru(bpy)_3_^2+^ oxidation reached its peak value and at the same time the ECL signal reached the highest (∼1422 RLU, see curve a and b in Fig. 3B). To evaluate the ECL behavior of Ru(bpy)_3_^2+^ and TPrA system under the transient potential (TP) excitation, the ECL signal was recorded every 10 ms for 100 ms. The dots in the curve c of Fig. 3B represented the ECL light signal under different TP excitation. In the optimal excitation potential optimization experiment, to eliminate the effect of TPrA's concentration, the mixture of Ru(bpy)_3_^2+^ and TPrA was replaced after each measurement. The PVD microchip #4 was utilized with a transient potential (TP) ranging from 1.5 V to 2.2 V, resulting in increased light signal intensity as the TP increased. By further increasing the TP value, a continuous increase in the ECL signal was found. When the TP continued to increase (>2.6 V), the ECL signal became reduced. The ECL signal (∼12 138 RLU) under the optimal TP (∼2.6 V) excitation was about eight times as large as the peak intensity of ECL signal (∼1422 RLU) during LSV process. Therefore, strong ECL signal from Ru(bpy)_3_^2+^ and TPrA system in a two-electrodes based microchip can be initiated by TP excitation, but the best TP value is not consistent with the peak potential obtained from the oxidative process by LSV.

### 3.3 Effect of electrodes' size on the ECL behavior of Ru(bpy)_3_^2+^/TPrA with microchips of two-electrode system

ECL signal of Ru(bpy)_3_^2+^/TPrA system stimulated by an optimal TP can be much higher than that from the LSV scanning. While the optimal potential may be not consistent with the peak potential of LSV scanning, the best working voltage for Ru(bpy)_3_^2+^/TPrA system on microchips should be measured with the potentiostatic method. With the mixture of 0.1 μM Ru(bpy)_3_^2+^ and 30 mM TPrA, we tested the optimal voltage and corresponding ECL intensity on SPE chip with Au & Au electrodes and PVD chips with Au & Au electrodes, respectively. The results were shown in Fig. 3C. For two-electrode based SPE chip, the optimal voltage of TP stimulation for ECL assay was 2.1 V, which was the same as that of LSV scanning excitation (see curve #3 in Fig. 3C). Compared to SPE chip #3, the PVD chip, with the same size as WE and CE, exhibited a higher optimal voltage (∼2.5 V) and a slightly enhanced ECL intensity (improved by 8.8%) (see curve #3, and #8 in Fig. S1†). With the size of WE remaining unchanged, as the distance between WE and CE was reduced to 200 μm, the optimal working voltage decreased to 2.4 V. This resulted in a 6.1% increase in ECL intensity (see curve #9 in Fig. S1†). Compared with PVD chip #9, the distance between WE and CE in PVD chip #4 was the same, but the area of WE in PVD chip #4 decreased and the optimal voltage for ECL assay increased to 2.6 V, while the intensity of ECL signal for PVD chip #4 decreased by 33.8% (see curve #4 in Fig. 3C). In addition, it was found that the ECL reaction of three kinds of PVD chips with the similar structure had certain regularity. The peak potential of ECL signal in PVD chip #4, #5 and #6 was 2.6 V, 2.7 V or 2.9 V (see curve #4, #5 and #6 in Fig. 3C). A constant distance between the WE and CE, when coupled with a larger WE area, led to a lower optimal working voltage and higher luminous intensity (see curve #4, #5 and #6 in Fig. 3C).

To validate the effect of distance between WE and CE on ECL assay, PVD chips with identical WE area (a 900 μm radius of WE) but varying sizes of CE were employed for the ECL test. As shown in Fig. S2,† as the distance decreased from 750 μm to 500 μm, the optimal voltage for the ECL assay dropped from 2.7 V to 2.6 V, resulting in a slight increase in the ECL signal (an improvement of 1.5%, see curve #11, and #12 in Fig. S2†). Then the distance continued to reduce, the optimal voltage remained constant with a minimal improvement in ECL intensity (see curve #4, #10, and #11 in Fig. S2†). Compared with the area of the WE, the distance between the WE and CE in the PVD chip had a relatively low impact on the ECL assay. Herein, the results demonstrated PVD chip with circular integrated electrodes can be applied for ECL detection and has the similar performance with the SPE chip.

For another PVD chip with interdigital electrode system, the intensity of ECL signal become stronger with the increasing of TP. While the potential exceeded 2.2 V, some bubbles appeared on the surface of the electrodes due to the electrolysis of water in the solution (see curve #7 in Fig. 3C). Therefore, we chose 2.2 V as the best TP for PVD chip # 7. The interdigital electrode system in PVD hip #7 has a large relative area between WE and CE, achieving a highly efficient mass transfer.^27^ Therefore, the light emitted from the ECL system on interdigital electrode was the strongest among all microchips with two-electrode system.

### 3.4 Effect of buffer's ionic concentration on the ECL behavior of Ru(bpy)_3_^2+^/TPrA for PVD chip with Au/Au electrodes

In previous reports, the concentration of PBS used as an ECL buffer varied, ranging from 10 mM to 0.1 M.^1,11,13,19^ The ionic concentration of ECL buffer may impact the ECL behavior of Ru(bpy)_3_^2+^/TPrA system. Of the DPV chips with circular integrated electrodes tested, chip #4 featuring a 900 μm radius WE required the lowest working potential and yielded the highest light signal in the ECL assay. Therefore, DVP chip #4 was chosen to test the impact of ECL buffer's ionic concentration on the ECL assay.

**Fig. 4:**
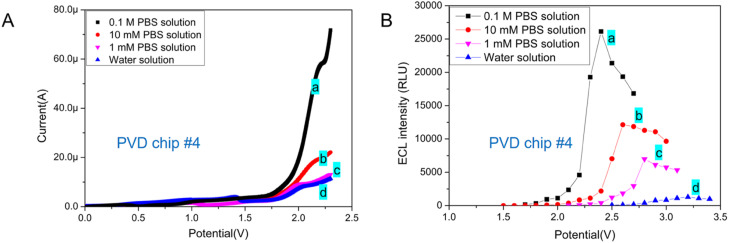
(A) Voltammograms (B) peak ECL intensity under different TP obtained at PVD chip #4 with a 900 μm radius of WE in (a) 0.1 M PBS solution (pH 7.0), (b) 10 mM PBS solution (pH 7.0), (c) 1 mM PBS solution (pH 7.0), and (d) water. All solutions contained 0.1 μM Ru(bpy)_3_^2+^ and 30 mM TPrA.

EC behavior of Ru(bpy)_3_^2+^/TPrA system in different buffer was tested on PVD chip #4. The LSV showed that the ionic concentration of reaction system has little impact on the oxidation peak potential of Ru(bpy)_3_^2+^ (Fig. 4A). As the increase of PBS concentration, the peak current increased obviously. In addition, the best working voltage under TP model was tested for ECL assay in buffers with different ionic concentration. From Fig. 4B, we can see that the increase of ionic strength in ECL system has improved the intensity of emitted light and reduced the optimal TP value. The concentrations of H_2_PO_4_^−^ and HPO_4_^2−^ in PBS buffer increased, resulting in the increase of deprotonation of TPrA^+^˙ and therefore a high concentration of Ru(bpy)_3_^2+^* and an improvement of ECL signal.^28,29^ Therefore, ionic concentration of buffer has significant effect on increasing the catalytic current of Ru(bpy)_3_^2+^/TPrA system and ECL signal in microchips with two electrodes.

In biochemical analysis, 10 mM PBS (1× PBS) is a commonly used buffer solution for diluting samples. Generally, 0.1 M PBS (10× PBS) is used for long-term storage. And the 10× PBS buffer is diluted to form a 1× PBS buffer for detection. For our further study, the PVD microchip based ECL detection method would be applied for biomarker (protein) detection. Therefore, 10 mM PBS was chosen to be used as ECL buffer for detection.

### 3.5 The detection of Ru(bpy)_3_^2+^ with microchips

**Fig. 5:**
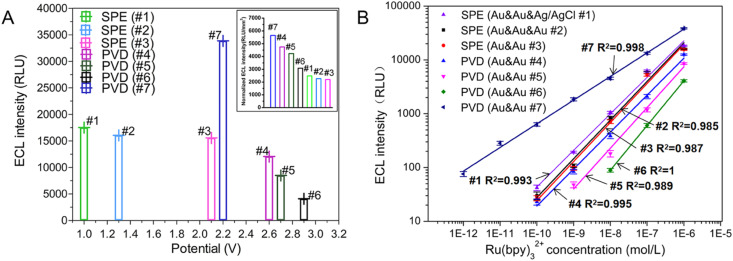
(A) Optimal potential and corresponding ECL signal for three SPE chips and four PVD chips in PBS solution (0.01 M, pH 7.0) containing 0.1 μM Ru(bpy)_3_^2+^ and 30 mM TPrA. The inset showed the normalized ECL intensity by the area of WE for the seven chips. (B) Calibration plots for the Ru(bpy)_3_^2+^ determination using different microchips under their optimal condition, respectively. The buffer is 0.01 M PBS solution, and the concentration of TPrA was 30 mM. Five replicate measurements were performed for all devices. Data were reported as average ± standard deviation. The *X* and *Y* axes were in a logarithmic scale to obtain a more condensed graphical representation.

To evaluate the feasibility of TP activation model for ECL assay, ECL signals under different TPs were detected in three SPE chips. The optimal working potential of TP stimulation for SPE chip#1, #2 and #3 were the same as the peak potential of LSV (Fig. 3A and 5A). Even though the best excited potential for SPE chip with Au & Au electrodes was larger than that of three-electrode electrochemical device, the intensity of ECL signal on SPE chip #3 kept similar with SPE chip #1 and #2. The results showed that similar ECL response of Ru(bpy)_3_^2+^/TPrA system could be observed on SPE chip without RE under an optimal TP.

The ECL behavior of Ru(bpy)_3_^2+^/TPrA system was measured with four PVD chips through TP activation. Because of the reduction in electrodes' size, the optimal working potentials for PVD chips with circular WE increased obviously compared to that of SPE chip. But the PVD chip with interdigital electrode has the similar optimal working potential to that of SPE chip. Four PVD chips with two electrodes exhibited higher normalized ECL responses compared to the SPE chips when the intensity was normalized by the area of WE (the data of area in Table 1). PVD chips with two-electrode system demonstrated larger optimal TP (2.2–2.9 V) for Ru(bpy)_3_^2+^/TPrA system compared to the screen-printed electrodes (SPE) chips with two-electrode system (∼2.1 V), leading to more than 39.9% improvement in normalized ECL signal intensity. The ECL response on interdigital electrodes was the strongest among all microchips (Fig. 5A inset).

To evaluate the linearity of Ru(bpy)_3_^2+^ on microchips, ECL responses were measured for different concentration of Ru(bpy)_3_^2+^ ranging from 1 pM to 1 μM on three SPE chips and four PVD chips, each using their respective optimal potential settings. As shown in Fig. 5B, the linear of Ru(bpy)_3_^2+^ for all SPE chips extended from 0.1 nM to 1 μM (see curve #1, #2, and #3 in Fig. 5B). The results revealed that RE was not indispensable for ECL assay in integrated microchips. Under the excitation of the optimal TP, microchips integrated with WE and CE can be applied for highly efficient ECL detection.

Furthermore, reducing the WE's radius from 1.5 mm to 900 μm did not significantly impact the ECL signal intensity observed on PVD chip #4. The linear relationship between logarithmic ECL intensity and logarithmic Ru(bpy)_3_^2+^ concentration was good in the range of 0.1 nM to 1 μM (*R*^2^ = 0.995, see curve #4 in Fig. 5B). As the radius of WE in PVD chip #5 was reduced to 800 μm, the sensitivity decreased to 1 nM. The sensitivity of PVD chip #6 was the lowest because the radius of WE was smallest (650 μm). For PVD chips #4, #5 and #6, there were four WE and a shared CE. Every two-electrode unit in one chip showed the similar ECL response when detecting Ru(bpy)_3_^2+^. While the PVD chip with interdigital electrode showed a highest ECL sensitivity of Ru(bpy)_3_^2+^ and a wide linear range between 1 pM to 1 μM (*R*^2^ = 0.998). An interdigital WE demonstrated higher catalytic efficiency for the Ru(bpy)_3_^2+^/TPrA system compared to a circular WE, resulting in improved sensitivity. To detect rare target molecule, microchips with interdigital electrodes may be a best choice for ECL assay. The rectangular shape of the WE and the small distance between the WE and CE make it difficult to selectively immobilize capture probes for ECL assays on the WE while excluding the CE using traditional modification methods. And the structure of interdigital electrode is not readily integrated with other units of interdigital electrode, limiting its application in multi-parameter detection.

For further application in detecting TPrA, PVD chip #4 was used to analyze different concentration of TPrA in 0.01 M PBS containing 10 μM Ru(bpy)_3_^2+^. As shown in Fig. S3,† the ECL intensity increased with the concentration of TPrA rose. There was a strong linear relationship between the logarithmic TPrA concentration and logarithmic ECL intensity across the range of 10 μM to 10 mM (*R*^2^ = 0.998, l g (ECL intensity) = 0.99 ± 0.02 l g (TPrA) +6.47 ± 0.08). Therefore, due to the good consistency and high sensitivity, the PVD microchip with two-electrode array can be applied for multi-parameter detection in further study.

## 4. Conclusions

In summary, the ECL behavior of Ru(bpy)_3_^2+^/TPrA system at microfluidic chips with integrated electrodes was investigated. The Ru(bpy)_3_^2+^/TPrA system exhibited strong ECL emission on microchips when an optimal transient potential (TP) was applied in the WE and CE without a RE. And the best TP for ECL assay on SPE chips were consistent with the peak potential by LSV scanning. While in PVD chip, the best TP for initiating the ECL reaction was larger than the peak potential by LSV scanning, but the ECL emission can reach eight times as strong as that peak ECL intensity excited by LSV model. The intensity of ECL can be improved by increasing the ionic concentration of ECL buffer. The effective electron transfer between interdigital WE and CE among PVD chip resulted in the highest ECL sensitivity, good linear relationship, wide detection scale and lower TP excitation. The good ECL response of Ru(bpy)_3_^2+^/TPrA system on a two-electrode system applied with a TP excitation suggested a possibility of designing ECL microchip without a RE for simultaneous multi-target detection.

## Author contributions

Haojun Yuan: methodology; project administration; writing – original draft; Baihui Liang: methodology; software; Ping Yang: investigation; writing – review & editing; Zhiwei Yang: methodology; software; supervision; Xinyi Cao: data curation; formal analysis; Yangbo Wu: methodology; supervision; Jie Zou: methodology; software; Qinghui Jin: resources; methodology; project administration; resources; writing – review & editing; Wanlei Gao: conceptualization; resources; project administration; supervision; writing – review & editing.

## Conflicts of interest

There are no conflicts to declare.

## Supplementary Material

RA-014-D3RA07298C-s001
