# Supplementary material for: Rapid and sensitive electrochemiluminescence detection using easily fabricated sensor with an integrated two-electrode system
Source: RSC Adv. 2024 Jan 19;14(5):3241–9. doi: 10.1039/d3ra07298c (PMC10797493; doi:10.1039/d3ra07298c)
Supplement: RA-014-D3RA07298C-s001 [file RA-014-D3RA07298C-s001.pdf]

Electronic Supplementary Material (ESI) for RSC Advances.
This journal is © The Royal Society of Chemistry 2024

Supplementary Material

Table S1. Relevant parameters of five PVD microchips.

Structure No.	Type	Electrode system	Working electrode size*	The area of Working electrode	Distance*	Microchip size
#8	PVD	Two-electrode	R=1.5 mm	7.065 mm^2	D=750 μm	12 mm×45 mm
#9	PVD	Two-electrode	R=1.5 mm	7.065 mm^2	D=200 μm	12 mm×45 mm
#10	PVD	Two-electrode	R=900 μm	2.543 mm^2	D=350 μm	13 mm×13 mm
#11	PVD	Two-electrode	R=900 μm	2.543 mm^2	D=500 μm	13 mm×13 mm
#12	PVD	Two-electrode	R=900 μm	2.543 mm^2	D=750 μm	13 mm×13 mm

* R means the radius of circular electrode. D means the distance between working electrode and counter electrode.

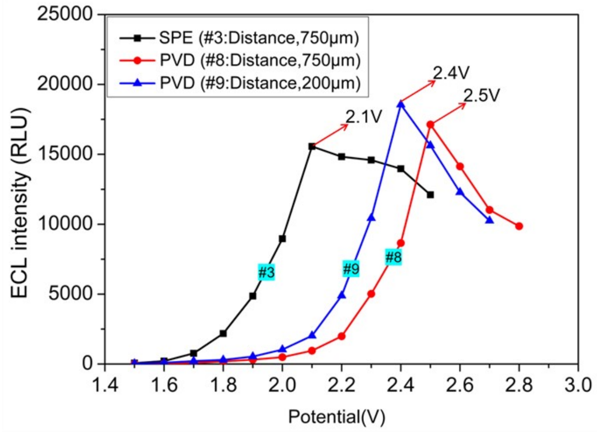

Fig. S1. ECL intensity from 0.1 μM $Ru(bpy)_3^{2+}$ and 30 mM TPrA in PBS solution (0.01 M, pH 7.0), achieved in SPE and PVD microchips with a 1500-μm radius of WE. Labeled value represents the distance between the counter electrode and working electrode for each microchip. The dots in each line represent ECL intensity during the measurement under different TP in the range of 1.5 V to 2.8 V.

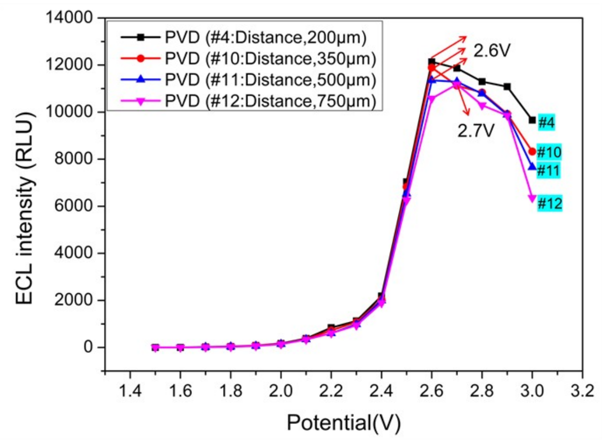

Fig. S2. ECL intensity from 0.1 μM Ru(bpy)$_3^{2+}$ and 30 mM TPrA in PBS solution (0.01 M, pH 7.0), achieved in PVD microchips with identical WE area (a 900-μm radius of WE) and different distance between WE and CE. The dots in each line represent ECL intensity during the measurement under different TP in the range of 1.5 V to 3.0 V.

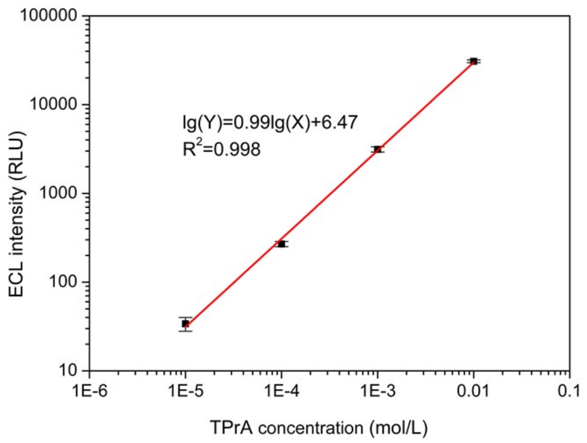

Fig. S3. Calibration plots for the TPrA determination using PVD chip (#4) with a 900-μm radius of WE. The buffer is 0.01 M PBS solution, and the concentration of Ru(bpy)$_3^{2+}$ was 10 μM. Five replicate measurements were performed for all devices. Data were reported as average ± standard deviation. The X and Y axes were in a logarithmic scale to obtain a more condensed graphical representation.
